# Quantitative proteomic dataset of the moss *Physcomitrium patens SMG1* KO mutant line

**DOI:** 10.1016/j.dib.2021.107706

**Published:** 2021-12-11

**Authors:** Anna Mamaeva, Anna Glushkevich, Igor Fesenko

**Affiliations:** Shemyakin-Ovchinnikov Institute of Bioorganic Chemistry of the Russian Academy of Sciences, Moscow, Russia

**Keywords:** Nonsense-mediated RNA decay, iTRAQ, Proteomics, *Physcomitrium patens*

## Abstract

Nonsense-mediated RNA decay (NMD) mechanism controls the quality of eukaryotic mRNAs by degradation of aberrant transcripts with a premature stop codon (PTC) in a pioneer round of translation. Besides aberrant transcripts, up to 10% of normal mRNA transcripts can be regulated by NMD. As NMD machinery is associated with translation, this system takes part in proteome formation in eukaryotic cells [1,2]. However, no proteomic datasets of plants with deficient NMD system are currently available.

Here, we provide an isobaric tag for relative and absolute quantitation (iTRAQ)-based quantitative proteomic dataset of the moss *Physcomitrium patens smg1* knockout line. The kinase SMG1 is one of the key components of the NMD system in many organisms, including plants. 8-day old protonema of wild type and mutant lines was used for the iTRAQ experiment in three biological replicates. LC-MS/MS data were processed using PEAKS Studio v.8 Software with protein identification based on a Phytozome protein database. Differentially expressed protein groups up- and down-regulated in the smg1 knockout line were found in the resulting dataset. Presented data can improve our understanding of NMD functions in plants.

## Specifications Table

**Table:** 

Subject	Omics: Proteomics
Specific subject area	Plant quantitative proteomics
Type of data	Table
How data were acquired	The raw data were acquired using Q Exactive HF mass spectrometer (Thermo Fisher Scientific). The data have been analyzed by PEAKS 8.0 software
Data format	Raw and analyzed data
Description of data collection	Protonemata of WT and *smg1Δ* lines were grown in 200 ml liquid BCD medium supplemented with 5 mM ammonium tartrate (BCDAT) during a 16 h photoperiod at 25 °C [3]. After 8 days, protonemata were collected for analysis. The experiment was performed in three biological replicates.
Data source location	Shemyakin-Ovchinnikov Institute of Bioorganic Chemistry of the Russian Academy of Sciences Moscow Russia
Data accessibility	Repository name: ProteomeXchange Data identification number: PXD029205 Direct URL to data: https://webi.ac.uk/pride/archive/projects/PXD029205

## Value of the Data

• The NMD machinery is directly coupled with the translation process. Transcriptomes of plants with knockouts or knockdowns of NMD components are available for a number of plant species. The presented data is the first quantitative proteomics dataset of the NMD-deficient plant.
• *Physcomitrium patens*, unlike another model plant *Arabidopsis thaliana*, has a most common type of NMD system that includes SMG1 kinase. It makes this plant a suitable model for NMD research [4]. Therefore, the presented data can be used to study NMD functions in eukaryotes.
• The presented data can be reanalyzed using available transcriptomic datasets of *P. patens* [4,5] to expand the range of identified NMD-regulated proteins and peptides. Using both transcriptome and proteome data can improve our understanding of NMD functions in plants.

## 1 Data Description

**Fig. 1:**
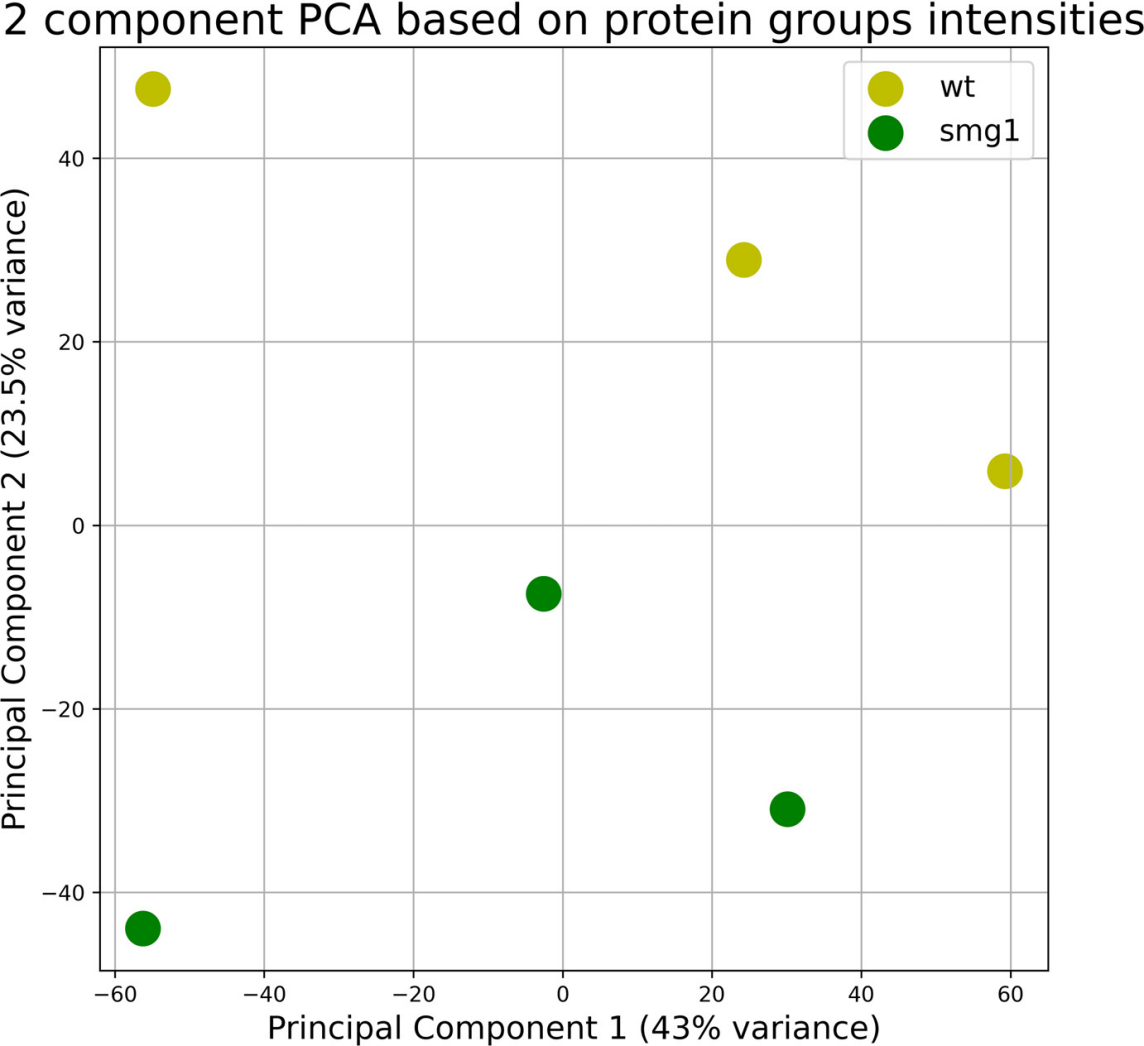
Principal component analysis (PCA) scatter plot of wild type (yellow) and *smg1* (green) samples. The PCA analysis included all protein groups and was performed in the Python library sklearn.

The presented data is a result of comparative quantitative proteomic analysis of the moss *Physcomitrium patens* SMG1 KO mutant line 2 [4]. We used isobaric tags for relative and absolute quantitation (iTRAQ) for an accurate study of quantitative proteomic changes in *smg1* plants in comparison to wild-type plants. The 8-day old protonemata were collected and used for the experiments. The raw LC-MS/MS data can be found in the ProteomeXchange database, accession number PXD029205. Further analysis with PEAKS 8.0 Studio Software identified 19049 peptides, which were assigned to 4308 protein groups (Table S1). Table S1 includes the information about iTRAQ intensity values of identified proteins and statistically significant differences between samples. Principal component analysis based on intensities of all identified protein groups is shown in Fig. 1.

Because iTRAQ quantification underestimates the amount of real fold change [6], fold change ratios thresholds of ⩾ 1.20 or ⩽ 0.83 (*P*  < 0.01, one-way ANOVA) were used to identify differentially expressed protein groups (DEPs). Overall, 105 downregulated and 36 upregulated protein groups were identified. GO terms enriched in differentially expressed proteins (Table S2) were found with g:Profiler tool [7].

## 2 Experimental Design, Materials and Methods

### 2.1 Plant material

The moss *Physcomitrium patens* subsp. *patens* (“Gransden 2004”, Freiburg) of wild type and SMG1 KO mutant line 2 produced by James P. B. Lloyd [8] were used in this study. The moss protonemata were grown in 200 ml liquid BCD medium supplemented with 5 mM ammonium tartrate (BCDAT) during a 16 h photoperiod at 25 °C for 8 days [3].

### 2.2 Protein extraction and trypsin digestion

Proteins were extracted with the phenol extraction method [9]. Samples were homogenized in ice-cold extraction buffer (500 mM Tris–HCl, pH 8.0, 50 mM EDTA, 700 mM sucrose, 100 mM KCl, 1 mM PMSF, 1 mM DTT), followed by 10 min incubation on ice. An equal volume of ice-cold Tris–HCl (pH 8.0)-saturated phenol was added, and the mixture was vortexed and incubated for 10 min with shaking. After centrifugation (10 min, 5500 × g, 4 °C), the phenol phase was collected and re-extracted with an extraction buffer. Proteins were precipitated with four volumes of ice-cold 0.1 M ammonium acetate in methanol overnight at −20 °C. The samples were centrifuged (10 min, 5500 × g, 4 °C) and pellets were rinsed with centrifugation (10 min, 5500 × g, 4 °C) by ice-cold 0.1 M ammonium acetate in methanol three times and with ice-cold acetone once. The resulting pellets were dried and dissolved in 8 M urea, 2 M thiourea and 10 mM Tris. Proteins were quantified by Bradford protein assay (Bio-Rad, Hercules, CA USA). The 100 µg of proteins were reduced by 5 mM DTT for 30 min at 50 °C and alkylated by 10 mM iodoacetamide for 20 min at room temperature. Proteins were dissolved in 40 mM ammonium bicarbonate and digested by 1 ug sequence-grade modified trypsin (Promega, Madison, WI, USA) at 37 °C overnight. The reaction was stopped by adding trifluoroacetic acid to the final concentration of 1%. 20 µg of each sample was desalted by Empore octadecyl C18 extraction disks (Supelco, USA) and then was dried in a vacuum concentrator. iTRAQ labelling (Applied Biosystems, Foster City, CA, USA) was conducted according to the manufacturer's manual. Proteins were labelled with the iTRAQ tags as follows: Wild type – 113, 115, 119 isobaric tags; SMG1 KO plants – 114, 116, 121. Samples were mixed, vacuum dried and dissolved in 100 μL 0.5% formic acid. The mixture was fractionated on SCX extraction disks (Supelco, USA): fractions were consistently eluted by 50, 75, 125, 200, 300 mM ammonium acetate in 20% acetonitrile supplemented with 0.5% formic acid; 5% ammonium hydroxide in 80% acetonitrile and 10% ammonium hydroxide in 60% acetonitrile. Each fraction was dried in a vacuum concentrator, dissolved in 80 μL 0.1% trifluoroacetic acid and desalted using Empore octadecyl C18 extraction disks (Supelco, USA) and dried in a vacuum concentrator. Samples were dissolved in 3% acetonitrile with 0.1% trifluoroacetic acid.

### 2.3 Liquid chromatography and mass spectrometry

LC-MS/MS analysis was conducted as described in our previous studies [10,11]. Peptides were separated on Acclaim PepMap 100 C18 (75 × 50 cm) (Thermo Fisher Scientific). Reverse-phase chromatography was performed with an Ultimate 3000 Nano LC System (Thermo Fisher Scientific), which was coupled to the Q Exactive HF benchtop Orbitrap mass spectrometer (Thermo Fisher Scientific) via a nanoelectrospray source (Thermo Fisher Scientific). Peptides in 5 ml of loading buffer (3% (vol/vol) acetonitrile, 0.1% (vol/vol) TFA in Milli-Q deionized water) were loaded on a trapping column PepMap 100 C18 (0.1 × 20 mm) (Thermo Fisher Scientific) at a flow rate of 5 ml/min for 6 min. NanoLC pump mobile phases were: A – (2% (vol/vol) acetonitrile, 0.1% (vol/vol) formic acid in Milli-Q deionized water; B - (80% (vol/vol) acetonitrile, 0.1% (vol/vol) formic acid, 19.9% (vol/vol) milli-Q deionized water. Peptides were eluted from the trapping column with a linear gradient: 5–28% B for 90 min; 28–45% B for 20 min, and 45–100% B for 7 min at a flow rate of 350 nL/min. After each gradient, the column was washed with 100% buffer B for 5 min and reequilibrated with buffer A for 10 min. Peptides were analyzed on a mass spectrometer, with one full scan (375–1400 m/z, R = 120,000 at 200 m/z) at a target of 3*10^6^ ions and max ion fill time 50 ms, followed by up to 15 data-dependent MS/MS scans with higher-energy collisional dissociation (HCD) (target 1*10^5^ ions, max ion fill time 100 ms, isolation window 1.2 m/z, normalized collision energy (NCE) 32%), detected in the Orbitrap (*R* = 30,000 at fixed first mass 100 m/z). Other settings: charge exclusion – unassigned, 1, > 6; peptide match – preferred; exclude isotopes – on; dynamic exclusion – 60 s was enabled.

### 2.4 Protein identification and quantification

Tandem mass spectra were analysed by PEAKS Studio version 8.0 software (Bioinformatics Solutions Inc., Waterloo, Canada) [12]. The custom database was built from the Phytozome database *P. patens* combined with chloroplast (85 entries) and mitochondrial (42 entries) proteins (87,142 records). From 87,015 protein nuclear-coding isoforms, 65,648 contained annotated domains. The database search was performed with the following parameters: a fragmentation mass tolerance of 0.05 Da; parent ion tolerance of 10 ppm; fixed modification – carbamidomethylation; variable modifications – oxidation (M) and acetylation (Protein N-term). The resulting protein list was filtered by a 1% false discovery rate (FDR).

PEAKS Q was used for iTRAQ quantification. Normalization was performed by averaging the abundance of all peptides. Median values were used for averaging. Given that iTRAQ quantification typically underestimates the degree of real fold changes between two samples, differential protein screening was performed using a fold change ratio ⩾ 1.20 (for upregulated DEPs) or ⩽ 0.83 (for downregulated DEPs) and Significance ⩾ 15.

## Ethics Statement

This work didn't involve human subjects, animal experiments and data collected from social media platforms.

## CRediT authorship contribution statement

**Anna Mamaeva:** Investigation, Writing – original draft. **Anna Glushkevich:** Writing – original draft, Visualization. **Igor Fesenko:** Formal analysis, Writing – review & editing, Data curation.

## Declaration of Competing Interest

The authors declare that they have no known competing financial interests or personal relationships which have, or could be perceived to have, influenced the work reported in this article.

## Appendix Supplementary materials

Image, application 1

Image, application 2
